# A Novel Glycolysis-Related Four-mRNA Signature for Predicting the Survival of Patients With Breast Cancer

**DOI:** 10.3389/fgene.2021.606937

**Published:** 2021-01-28

**Authors:** Xiaolu Zhang, Jia Wang, Jing Zhuang, Cun Liu, Chundi Gao, Huayao Li, Xiaoran Ma, Jie Li, Changgang Sun

**Affiliations:** ^1^College of First Clinical Medicine, Shandong University of Traditional Chinese Medicine, Jinan, China; ^2^College of Traditional Chinese Medicine, Shandong University of Traditional Chinese Medicine, Jinan, China; ^3^Department of Oncology, Weifang Traditional Chinese Hospital, Weifang, China; ^4^Qingdao Academy of Chinese Medical Sciences, Shandong University of Traditional Chinese Medicine, Qingdao, China

**Keywords:** glycolysis, breast cancer, prognosis, signature, univariate and multivariate Cox analyses

## Abstract

**Background:** Glycolysis is critical in the occurrence and development of tumors. Owing to the biological and clinical heterogeneity of patients with BRCA, the traditional predictive classification system is far from satisfactory. Survival and prognosis biomarkers related to glycolysis have broad application prospects for assessing the risk of patients and guiding their individualized treatment.

**Methods:** The mRNA expression profiles and clinical information of patients with BRCA were obtained from TCGA database, and glycolysis-related genes were obtained by GSEA. Patients with BRCA were randomly divided into the training cohort and testing cohort. Univariate and multivariate Cox analyses were used to establish and validate a new mRNA signature for predicting the prognosis of patients with BRCA.

**Results:** We established a four-gene breast cancer prediction signature that included *PGK1, SDHC, PFKL*, and *NUP43*. The patients with BRCA in the training cohort and testing cohort were divided into high-risk and low-risk groups based on the signature. The AUC values were 0.74 (training cohort), 0.806 (testing cohort) and 0.769 (entire cohort), thereby showing that the prediction performance of the signature is acceptable. Additionally, Cox regression analysis revealed that four-gene signature could independently predict the prognosis of BRCA patients without being affected by clinical factors.

**Conclusion:** We constructed a four-gene signature to predict the prognosis of patients with BRCA. This signature will aid in the early diagnosis and personalized treatment of breast cancer, but the specific associated biological mechanism requires further study.

## Introduction

The Warburg effect has become a hallmark of cancer, and it is considered to be a very promising target for cancer treatment (Tekade and Sun, 2017). When the oxygen supply is sufficient, normal cells convert glucose to pyruvate, which is phosphorylated during mitochondrial oxidation to produce ATP; under anaerobic or hypoxic conditions, cells use fermentation to convert glucose to lactic acid (Wong et al., 2020). However, even without lack of oxygen, glucose is also converted to lactic acid by tumor cells, and the metabolic characteristics of this aerobic glycolysis is called the Warburg effect, which has been targeted in the development of imaging and therapeutic drugs. For example, 2-deoxy-2-[^18^F]fluoro-glucose (^18^F-FDG) has been reported to respond well to the glucose metabolism in various organs and tissues in the body, and PET with ^18^F-FDG is widely used clinically. To date, many glycolytic genes/proteins have been found to be abnormally expressed in tumors and are crucial in tumor development and progression, and these genes and proteins can be used as targets for tumor diagnosis or treatment. For instance, the overexpression of glucose transporter 1 (*GLUT1*) in head and neck squamous cell carcinoma (HNSCC) leads to the activation of the NFκB signaling pathway, which is related to a low survival rate in HNSCC patients (Li et al., 2013). Additionally, *GLUT1* has been found to be overexpressed in kidney cancer (Chan et al., 2011), liver cancer (Amann et al., 2009), ovarian cancer (Semaan et al., 2011), and other cancers. In oral squamous cell carcinoma (OSCC), lactate dehydrogenase A (*LDHA*) can facilitate glycolysis and epithelial-mesenchymal transition, thereby promoting the invasion and proliferation of OSCC cells (Cai et al., 2019). As a key rate-limiting enzyme in glycolysis, Pyruvate kinase M2 (*PKM2*) can catalyze the conversion of phosphoenolpyruvate to pyruvate. For gastrointestinal tumors, *PKM2* is regarded as a useful target to aid in diagnosis, screening, and treatment (Guo et al., 2018). Knockout of *PKM2* can increase the radiosensitivity of cervical cancer (CC) cells and is considered to be an important target for enhancing the radiosensitivity of patients with CC (Lin et al., 2019). A clearer understanding of the mechanism of glycolysis in cancer may help guide more specific treatment strategies.

Glycolysis has also been shown to act a pivotal part in the occurrence and development of BRCA. Han et al. reported that the overexpression of *PRMT5* regulated the LXRα/NF-κBp65 pathway, thereby promoting the proliferation, invasion, and aerobic glycolysis in breast cancer cells (Han et al., 2020). Under hypoxic conditions, the silencing of *circDENND4C* was found to increase *Mir-200b/c*, which inhibited glycolysis in breast cancer cells as well as their migration and invasion (Ren et al., 2019). The hypoxia-induced oxidation of ATM promotes the glycolysis activity of cancer-associated fibroblasts (CAFs), and lactic acid produced by hypoxia CAFs can activate the TGFβ1/p38 MAPK/MMP2/9 signaling axis and promote the invasion and metastasis of breast cancer cells (Sun et al., 2019). Research on glycolysis may provide new information to better understand the occurrence and progression of BRCA and to better predict its prognosis. However, the current mechanism by which glycolysis-related genes function in breast cancer is not very clear. Therefore, it is of great importance to elucidate the relationship between glycolysis and breast cancer on the molecular level to increase the survival and prognosis of patients with BRCA.

In this study, we integrated high-throughput data and used univariate and multivariate Cox analyses to recognize new biomarkers associated with the survival and prognosis of breast cancer to aid in the development of new targeted therapies. We identified 256 mRNAs related to glycolysis and then established and validated a four-gene signature using Cox regression analysis. Notably, this glycolysis-related risk signal can be used to accurately predict a poor prognosis for patients with breast cancer. These outcomes supply a new perspective for breast cancer study.

## Materials and Methods

### Patient Genome Expression and Clinical Data Acquisition

The mRNA expression profiles and clinical information of patients with breast cancer were obtained from the Cancer Genome Atlas (TCGA) (https://cancergenome.nih.gov/), which is a public database that does not demand the approval of the local ethics committee. The clinical data included survival time, survival status, gender, age, TNM stage, and pathological stage. A total of 1,109 tumor samples and 109 normal samples were contained in this analysis.

### Screening of Glycolysis-Related Genes

We found five gene sets related to glycolysis in the Molecular Signatures Database v4.0 (http://www.broadinstitute.org/gsea/msigdb/index.jsp), including BIOCARTA_GLYCOLYSIS_PATHWAY, GO_GLYCOLYTIC_PROCESS, HALLMARK_GLYCOLYSIS, KEGG_GLY-COLYSIS_GLUCONEOGENESIS, and REACTOME_GLYCOLYSIS. Subsequently, Gsea_4.0.3 was used to detect whether there were dramatic differences in these five gene sets between breast cancer samples and normal samples (*P* < 0.05 was regarded as significant). The genes with significantly different gene sets were extracted for the next analysis.

### Functional Enrichment Analysis

The R module Profiler package was used to perform gene ontology (GO) and Kyoto Encyclopedia of Genes and Genomes (KEGG) enrichment analyses of glycolysis-related genes (*P* < 0.05). GO enrichment analysis was mainly used to describe the biological processes (BP), molecular functions (MF), and cellular components (CC) of genes. The KEGG analysis revealed the biological pathways of glycolysis-related genes.

### Construction and Analysis of a Protein-Protein Interaction (PPI) Network

We used the STRING database (http://string-db.org) to build a PPI network to explore the interaction between glycolysis-related genes. An interaction with a confidence score ≥ 0.9 was retained. Cytoscape software was used to visualize the network. The molecular complex detection (MCODE) in Cytoscape was used to perform a module analysis of the PPI network using the following default parameters: degree cut-off = 2, node score cut-off = 0.2, k-core = 2, and max. depth = 100. We applied the module Profiler package again for enrichment analysis of important modules.

### Construction and Validation of the Gene Signature

A total of 1,034 patients with BRCA were analyzed in terms of survival, excluding patients who were followed for <1 month. We used the R caret package to randomly divide the 1,034 patients into the training cohort and testing cohort. We performed univariate Cox analysis on the glycolysis genes previously screened in the training group to identify genes that were significantly (*P* < 0.05) related to overall survival (OS). Next, we used multivariate Cox regression analysis to determine prognostic-related genes and obtain correlation coefficients.

The following prognostic risk score formula was used:

Risk score = β gene1 × expr (gene 1) + β gene2 × expr (gene 2) +. + β genen × expr (gene *n*), where β represents the regression coefficient, which is obtained in the multivariate Cox analysis, and expr is the expression of the corresponding gene. We divided patients in the training cohort, testing cohort and entire cohort into high-risk group and low-risk group by using the median risk score as the threshold. R software was used to visualize the risk score distribution, survival status, and prognostic gene expression profile of patients in the training cohort. To evaluate the specificity and sensitivity of the multi-mRNA signature, we used the time-dependent receiver operating characteristic (ROC) curve and calculated the area under the curve (AUC).

### Validation of the Independence of the Multi-mRNA Signature

To further confirm the independence of the relationship between the multi-mRNA risk scores and clinical factors (consisting of age, pathological stage, and TNM stage) and the prognosis of patients with BRCA, we used R software to perform univariate and multivariate Cox analyses, and *P* < 0.05 was regarded as meaningful.

We also used the cBioPortal database (http://cbioportal.org) to analyze the genetic variation of the four genes in the signature.

## Results

### Extraction of Data on Glycolysis-Related Genes

**Figure 1:**
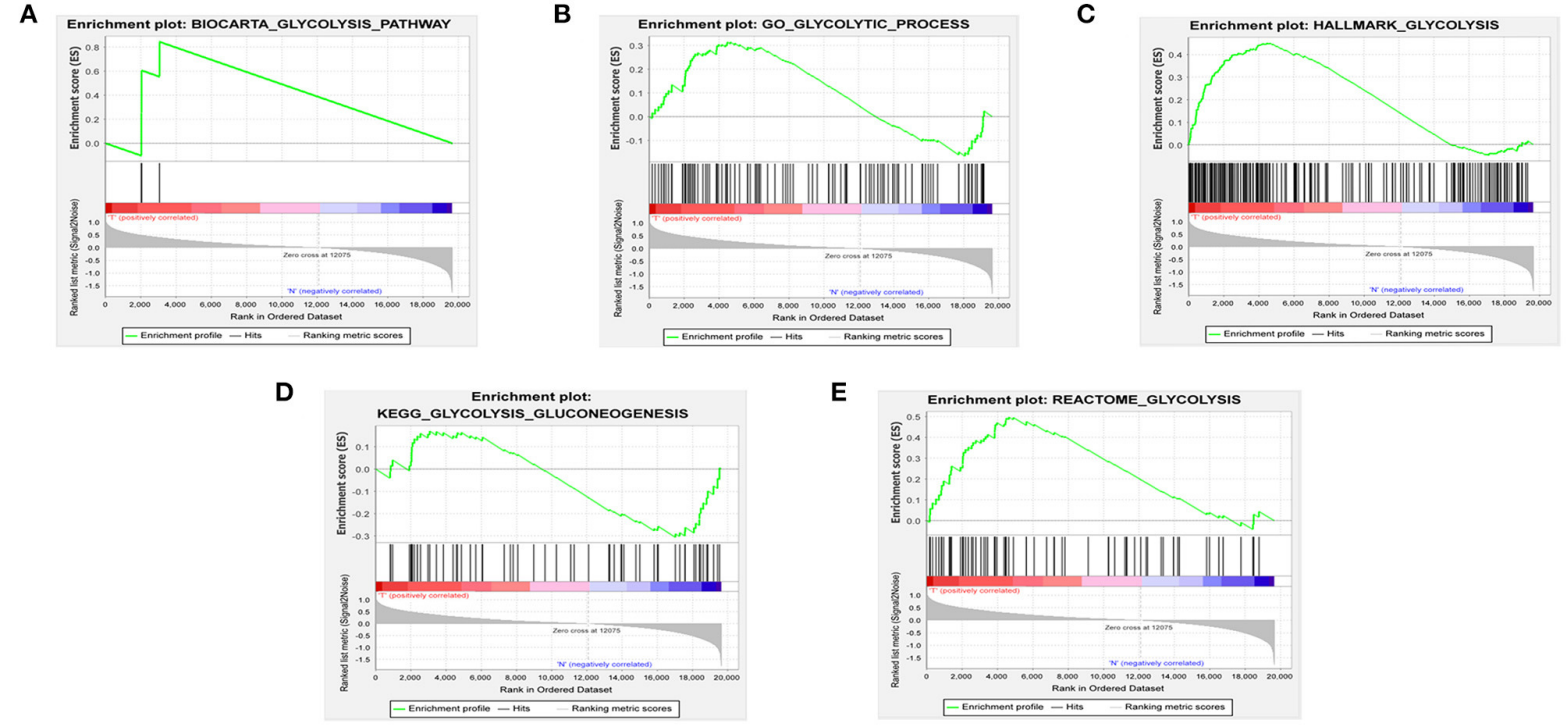
Enrichment of five glycolysis-related gene sets in GSEA. **(A)** BIOCARTA_GLYCOLYSIS_PATHWAY, **(B)** GO_GLYCOLYTIC_PROCESS, **(C)** HALLMARK_GLYCOLYSIS, **(D)** KEGG_GLYCOLYSIS_GLUCONEOGENESIS, **(E)** REACTOME_GLYCOLYSIS.

We obtained the mRNA expression profiles and clinical information of patients with BRCA from TCGA database. First, we used GSEA to study the differential expression of five gene sets related to glycolysis in tumor and normal samples. The effects indicated that there were dramatic differences between the tumor and normal samples in the HALLMARK_GLYCOLYSIS and REACTOME_GLYCOLYSIS gene sets (Figure 1). We then extracted data of a total of 256 glycolysis-related genes from these two gene sets.

### GO and KEGG Functional Enrichment Analyses

**Figure 2:**
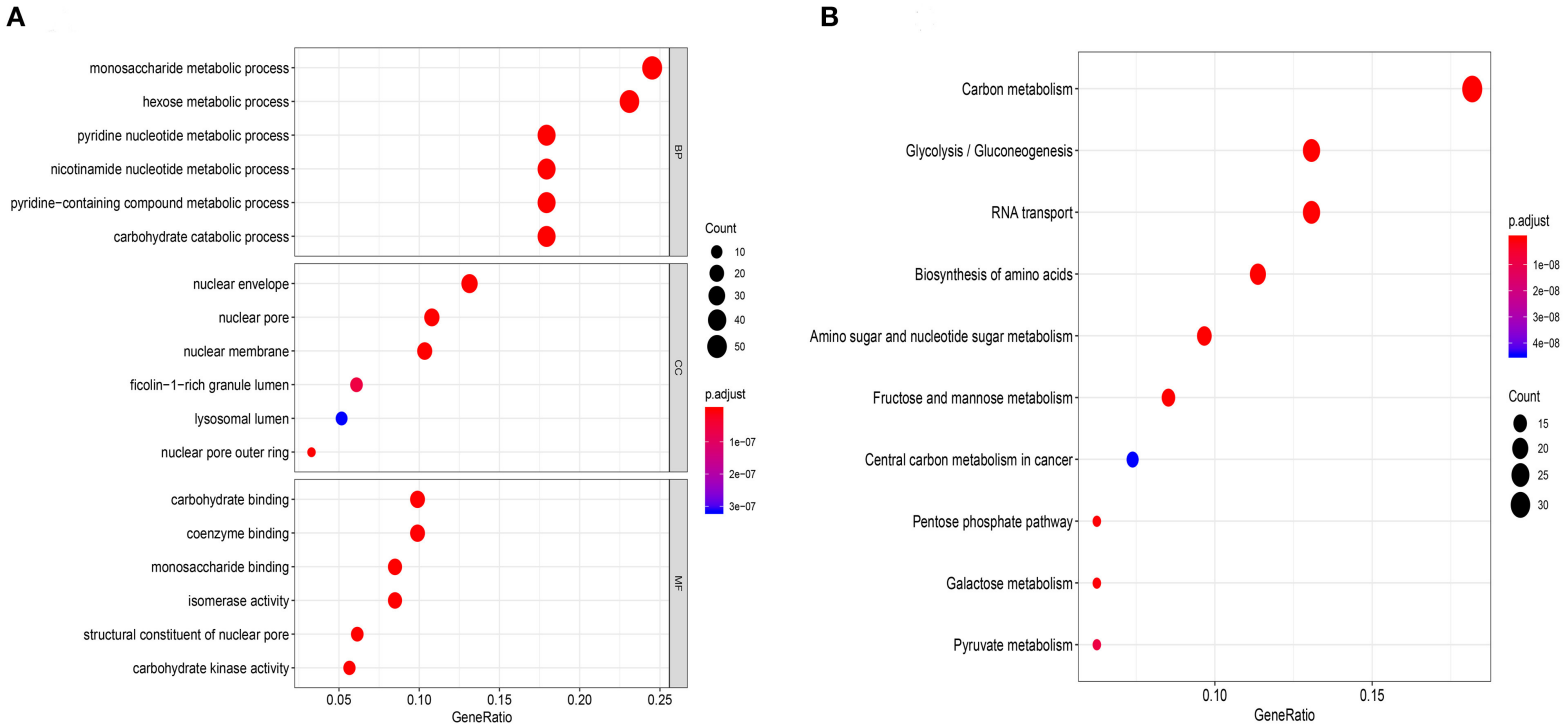
Functional enrichment analysis of glycolysis-related genes chose from GSEA. **(A)** Gene ontology biological process terms, **(B)** Enrichment of KEGG pathways. *P* < 0.05 was set as the cutoff criteria.

To verify whether these genes were related to glycolysis, we performed GO and KEGG enrichment analysis. The GO analysis revealed biological processes (BPs) related to metabolic processes involving monosaccharide and hexose (Figure 2A). The KEGG enrichment analysis revealed the involvement of carbon metabolism and glycolysis/gluconeogenesis (Figure 2B). These results indicated that the data extracted for the genes were indeed related to glycolysis.

### PPI Network Construction and Analyses on Module

**Figure 3:**
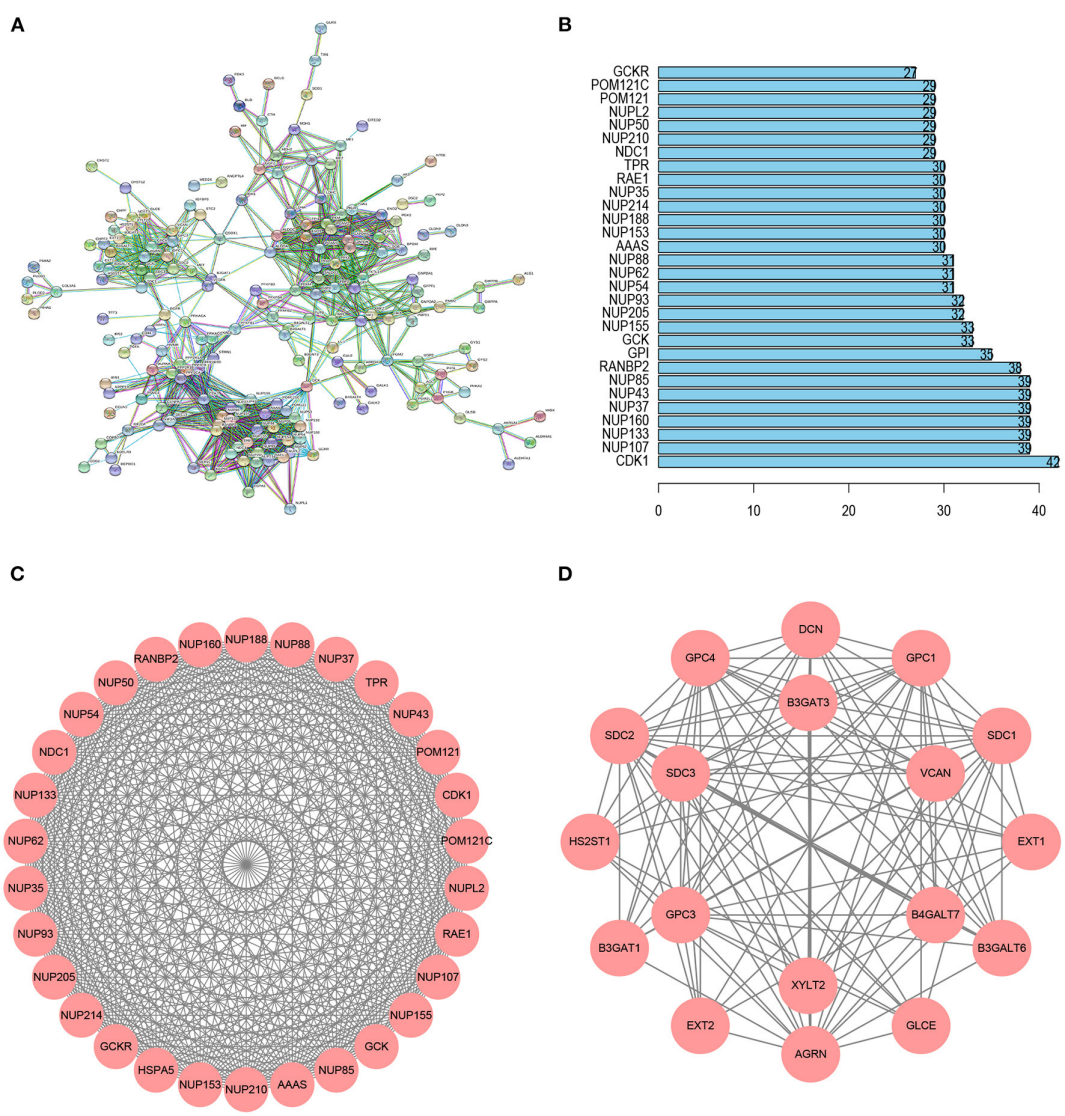
PPI network analysis of glycolysis-related genes. **(A)** The PPI network including 188 node and 1,157 edges. **(B)** Histogram of the top 30 genes in the PPI network. **(C)** Module 1 included 30 nodes and 430 edges with a score of 29.655. **(D)** Module 2 included 18 nodes and 114 edges with a score of 13.412.

Using the STRING database, we obtained a PPI network of glycolysis-related genes, which included 188 nodes and 1,157 edges (Figure 3A). The top 30 genes for connectivity are shown in Figure 3B. Among them, *CDK1* was the most important gene that contacted 42 nodes. A total of 11 modules were calculated based on MCODE in the Cytoscape software. The most important module, module 1, comprised 30 nodes and 430 edges (score = 29.655) (Figure 3C). Module 2 comprised 18 nodes and 114 edges (score = 13.412) (Figure 3D).

**Figure 4:**
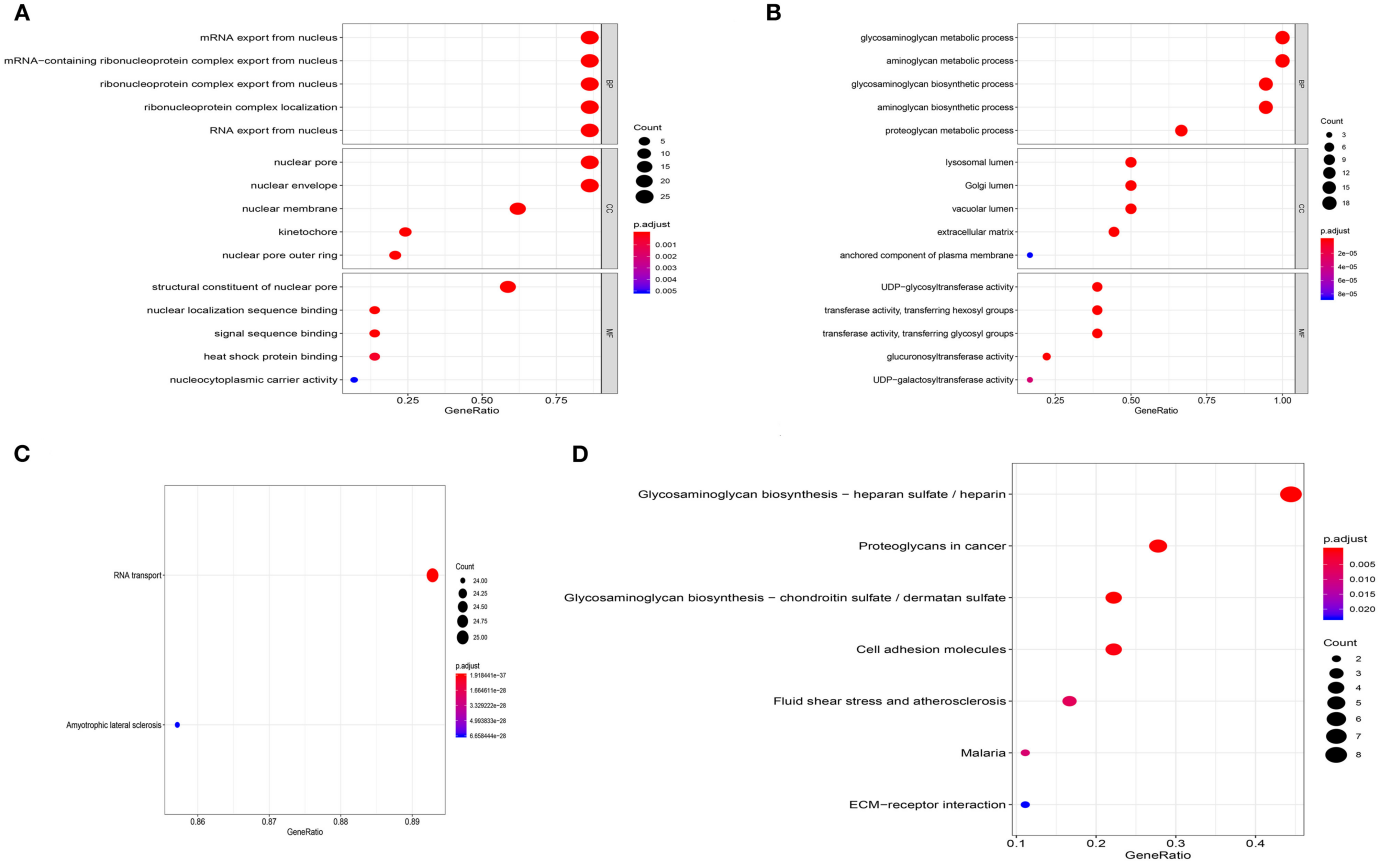
Enrichment analysis of the first two modules. **(A)** GO analysis of module 1. **(B)** GO analysis of module 2. **(C)** KEGG analysis of module 1. **(D)** KEGG analysis of module 2.

We carried out functional enrichment analysis for the genes of the first two modules. In the GO analysis, the genes in module 1 were mainly concentrated in mRNA export from the nucleus, nuclear pores, and structural constituents of nuclear pores (Figure 4A); in module 2, genes were mostly enriched in glycosaminoglycan metabolic processes, aminoglycan metabolic processes, lysosomal lumen, and UDP-glycosyltransferase activity (Figure 4B). The KEGG analysis outcomes indicated that in module 1, genes were mainly enriched in RNA transport and amyotrophic lateral sclerosis (Figure 4C); in module 2, genes were mainly concentrated in glycosaminoglycan biosynthesis–heparan sulfate/heparin, and proteoglycans in cancer (Figure 4D).

### Identification of Glycolysis-Related Genes Related to Survival and Prognosis of Breast Cancer

We randomly divided 1,034 breast cancer patients into the training cohort (*n* = 518) and testing cohort (*n* = 516). First, we performed univariate Cox regression analysis on the 256 glycolysis-related genes in the training cohort and screened out four genes (*PGK1, SDHC, PFKL*, and *NUP43*) related to OS (*P* < 0.01). Next, multivariate Cox analysis was performed to further screen genes associated with the survival and prognosis of patients with BRCA. Subsequently, these four genes were identified as prognostic-related genes (Table 1). Interestingly, these four genes showed relative risk with a hazard ratio (HR) > 1 and were associated with poor survival rates.

**Table 1:** Multivariate Cox regression analysis identified four glycolysis-related genes with respective coefficient.

**mRNA**	**coef**	**HR**	**HR.95L**	**HR.95H**	***P* value**
SDHC	0.0867	1.0906	1.0140	1.1730	0.0196
NUP43	0.0508	1.0521	1.0067	1.0995	0.0239
PFKL	0.0195	1.0197	0.9965	1.0434	0.0962
PGK1	0.0061	1.0061	1.0012	1.0110	0.0146

**Figure 5:**
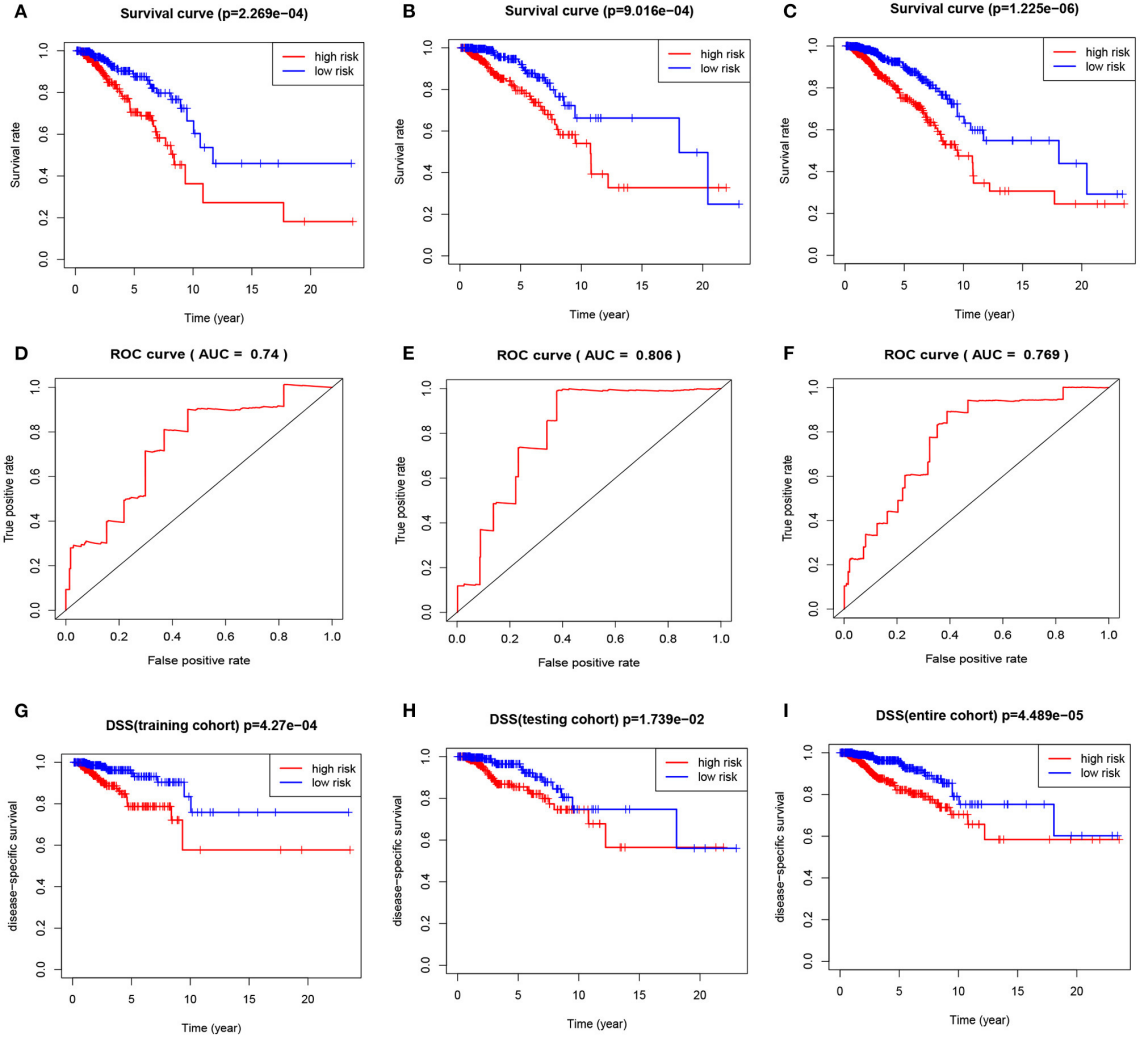
The four-mRNA signature predicts the prognosis of BRCA patients. **(A)** Kaplan-Meier curve of OS in the training cohort. **(B)** Kaplan-Meier curve of OS in the testing cohort. **(C)** Kaplan-Meier curve of OS in the entire cohort. **(D)** ROC curve of training cohort. **(E)** ROC curve of testing cohort. **(F)** ROC curve of entire cohort. **(G)** DSS curve of training cohort. **(H)** DSS curve of testing cohort. **(I)** DSS curve of entire cohort.

**Figure 6:**
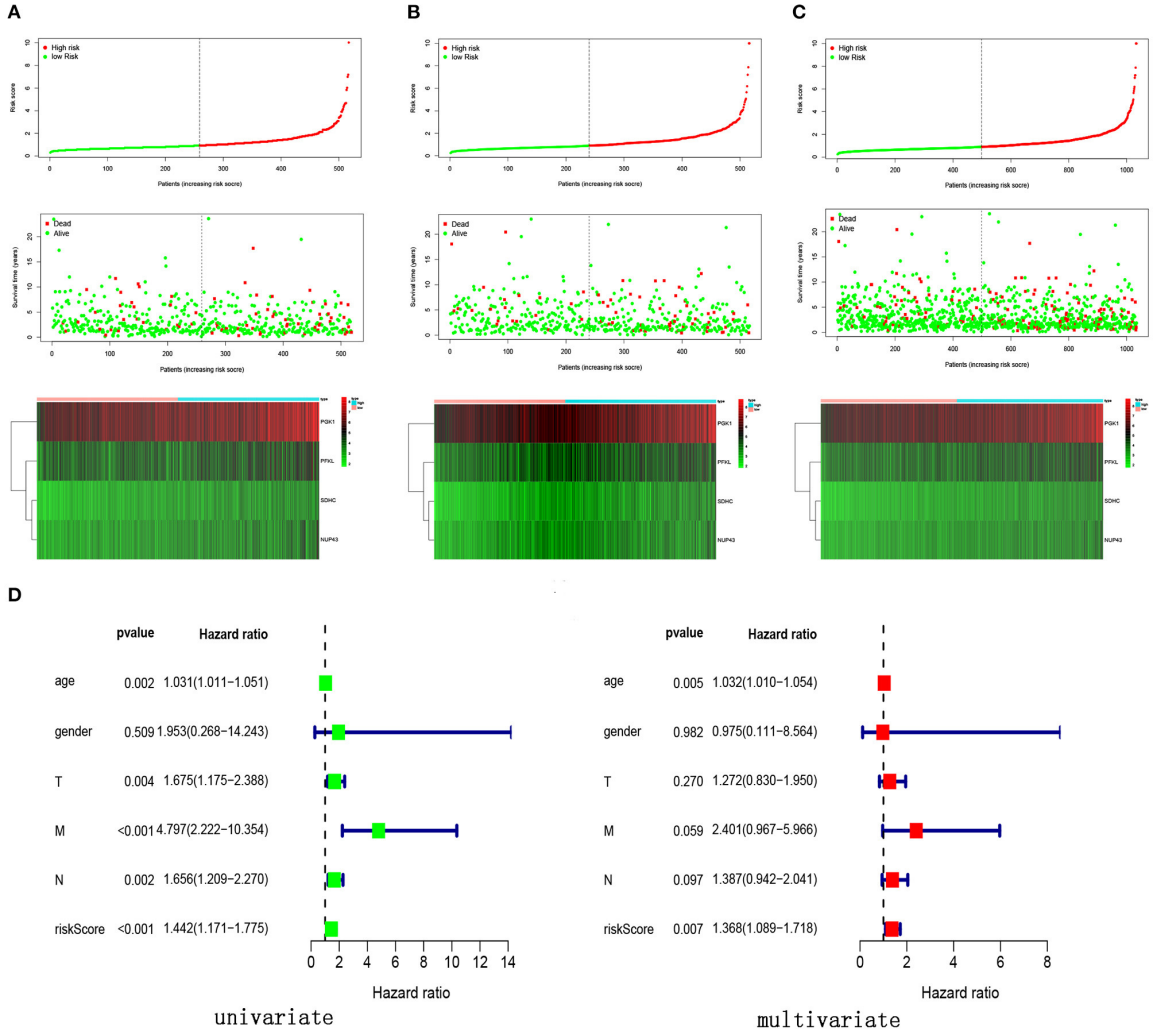
Risk score, survival status and expression levels of four glycolysis-related genes in different cohort. **(A)** Training cohort, **(B)** testing cohort, **(C)** entire cohort, **(D)** univariate and multivariate Cox regression analysis of the risk score and clinical feature in the training cohort.

We calculated the risk score for each patient by using the prognostic risk score formula. With the median risk score as the threshold, patients in the training cohort, testing cohort and entire cohort were divided into high-risk and low-risk groups. In these three groups, the Kaplan-Meier survival curve revealed that the survival rate of the high-risk group was significantly lower than that of the low-risk group (Figures 5A–C). As shown in Figures 5D–F, the AUC values of the dependent ROC for the four-mRNA signature were 0.74 (training cohort), 0.806 (testing cohort) and 0.769 (entire cohort), thereby indicating that the signature could predict the survival risk of patients with BRCA. We also analyzed disease-specific survival (DSS), and the results are shown in Figures 5G–I, which further illustrates the good performance of the four-mRNA signature. In addition, we have also drawn the risk plot of the training cohort (Figure 6A), testing cohort (Figure 6B) and entire cohort (Figure 6C), including risk scores for patients, the survival status of the patient, and the expression levels of the four genes included in the signature.

### Four-mRNA Signature Can Be Used as an Independent Prognostic Indicator of Breast Cancer

In order to verify the prognostic ability of four-mRNA signature can be independent of other clinical parameters, including age and TNM stage, univariate and multivariate Cox analyses were performed in the training cohort. The results showed that the four-mRNA prognosis signature was significantly associated with the patient survival rate (Figure 6D). Additionally, we found that age was also an independent prognostic factor with *P* values < 0.05 in univariate and multivariate Cox analysis.

### Analysis of the Four Genes in the Signature

**Figure 7:**
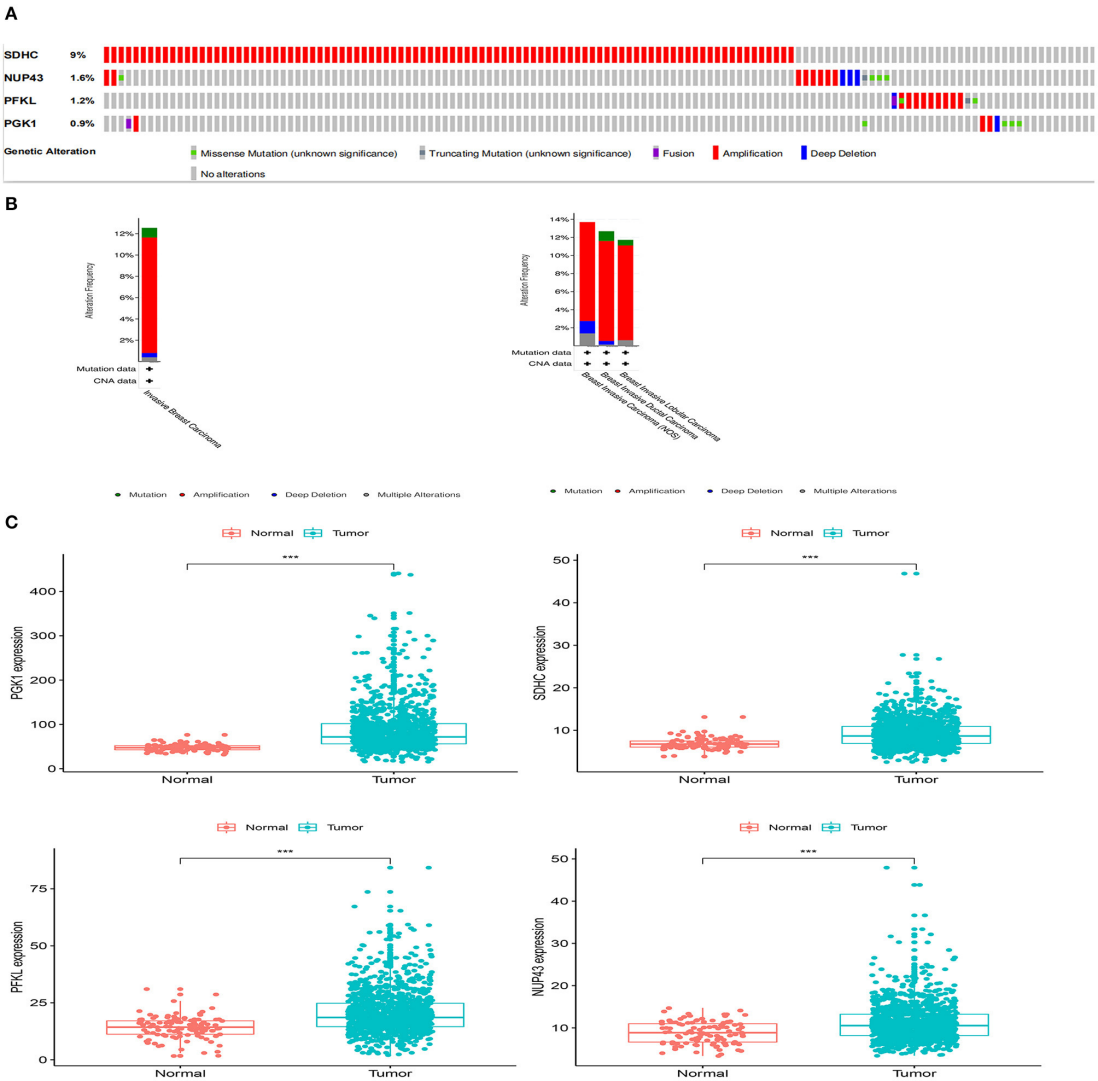
Gene mutation overview of four genes. **(A)** The four genes were altered in 125 (12.55%) of the 996 patients. **(B)** Specific alteration of selected genes in different pathological types of BRCA. **(C)** Differential expression analysis of the four selected genes.

We analyzed the genetic alterations of the four genes in the signature using the cBioPortal database, which consisted of data on 996 BRCA cases in TCGA database. The results indicated that 125 (12.55%) of the 996 patients had mutations. Among them, the *SDHC* gene was mutated in 9% of patients, the *NUP43* gene was mutated in 1.6% of patients, the *PFKL* gene was mutated in 1.2% of patients, and the *PGK1* gene was mutated in 0.9% of patients (Figure 7A).

In different types of cancer, specific changes in selected genes are also significant. In invasive breast carcinoma, 0.9% of the changes were mutations, 10.84% were amplifications, 0.4% were deep deletions, and 0.4% were multiple alterations. In breast invasive ductal carcinoma, breast invasive lobular carcinoma, and breast invasive carcinoma (NOS), the most common alteration was mutation (Figure 7B).

We aslo analyzed the differential expression of *PGK1, SDHC, PFKL*, and *NUP43* in tumor and normal samples. We found that the expression levels of these four genes were significantly upregulated in tumor specimens (Figure 7C).

### Establishment and Validation of a Prognostic Nomogram

**Figure 8:**
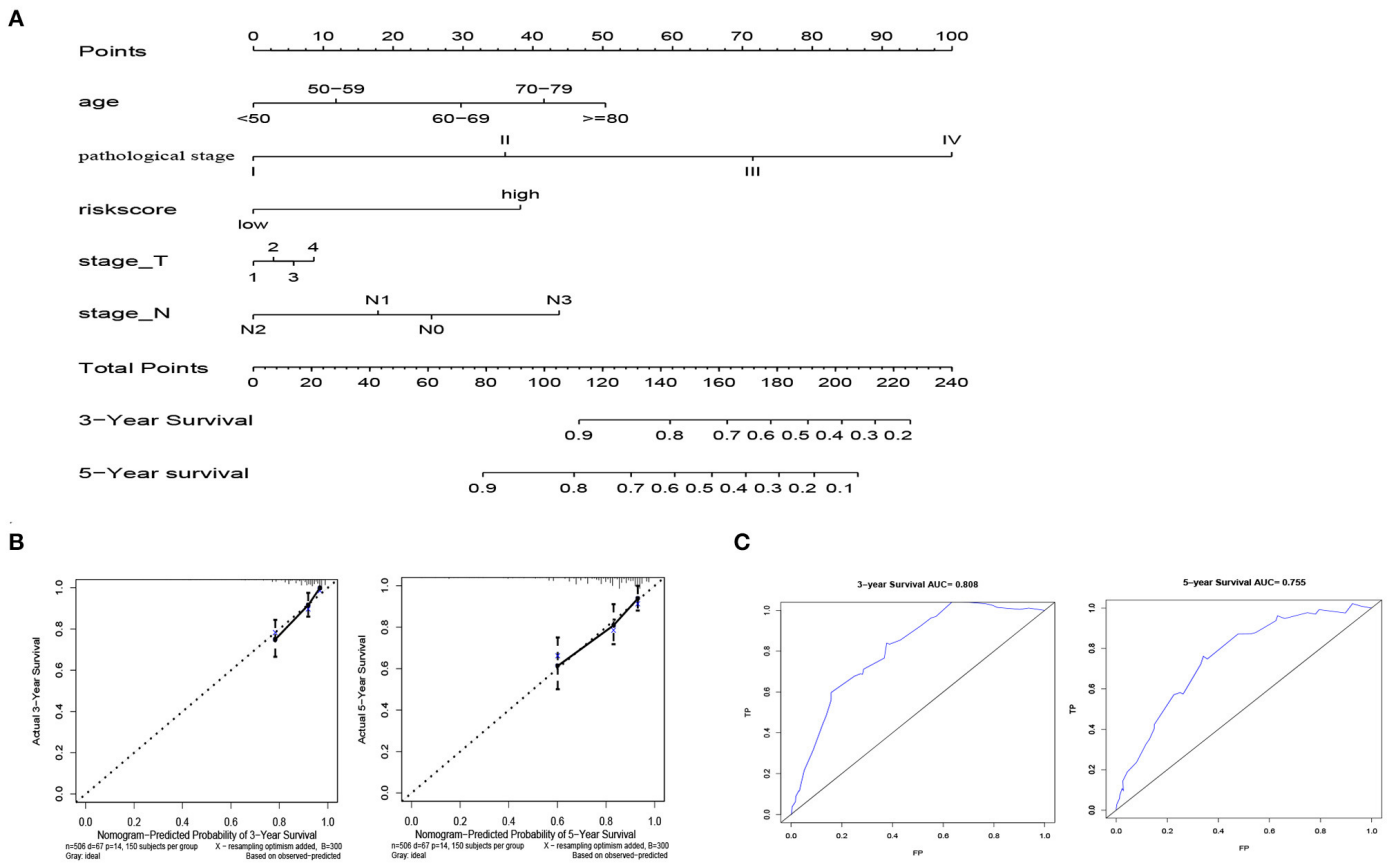
Establishment of nomogram model. **(A)** Normogram for predicting 3-year and 5-year OS in BRCA patients. **(B)**The calibration plots show that the nomogram has good predictability for the 3-year and 5-year OS of BRCA patients. **(C)** ROC curves of 3-year and 5-year OS prediction using the nomogram.

In order to facilitate clinical application, we established a nomogram that can predict 3-year and 5-year OS of BRCA patients based on 506 patients with complete clinical data in the training cohort. Risk score, age, pathological stage, T stage and N stage are included in the nomogram as parameters (Figure 8A). The calibration curve in Figure 8B showed that the predicted result of the nomogram is in good agreement with the actual situation. The AUC of the nomogram at 3-year and 5-year was 0.808 and 0.755, respectively (Figure 8C).

### Kaplan Meier Curve Was Used to Verify the Validity of Four-mRNA Signature for Survival Prediction

**Figure 9:**
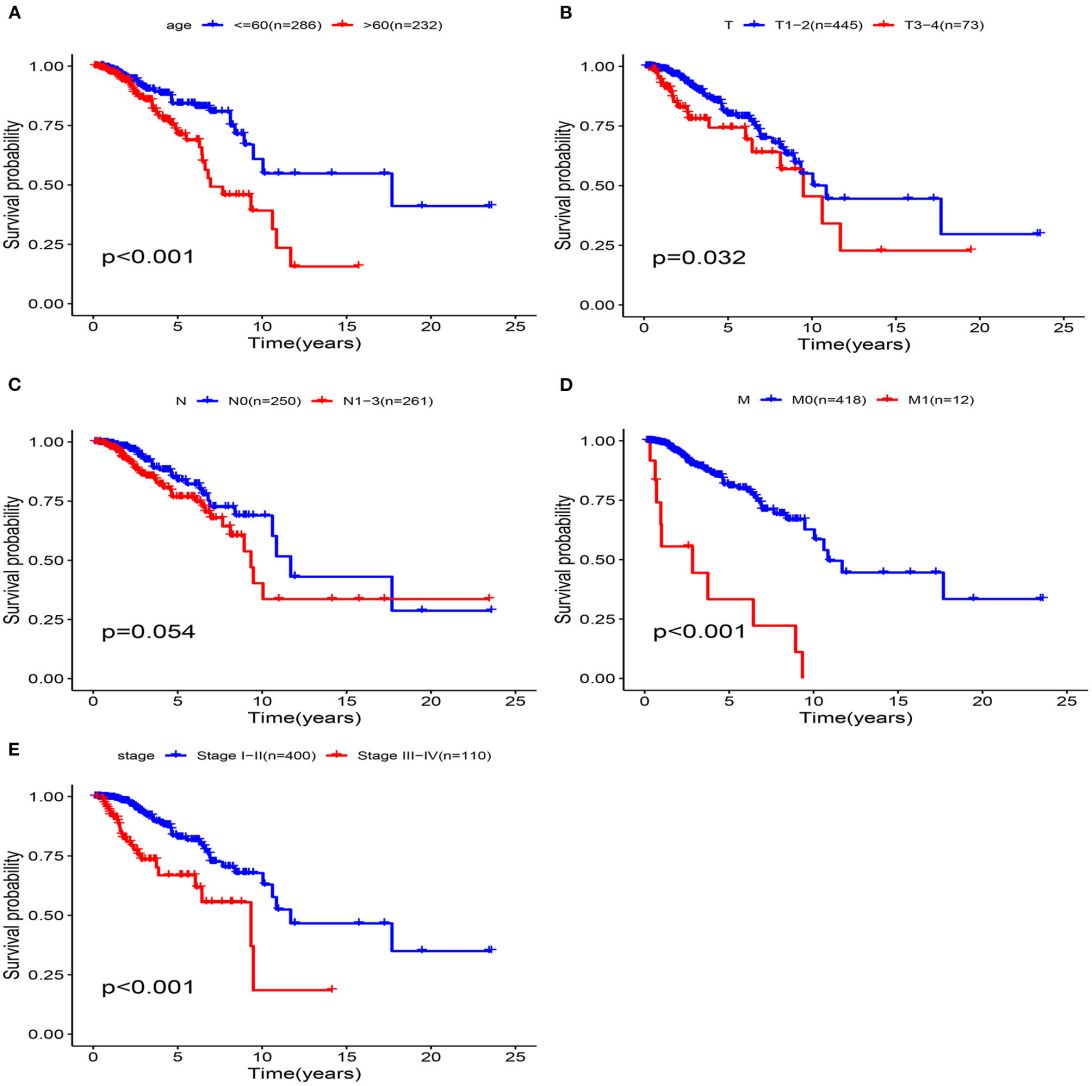
Clinical features predict patients survival in the training cohort. **(A)** Age, **(B)** T Stage, **(C)** N Stage, **(D)** M Stage, **(E)** pathological stage.

The univariate Cox analysis showed that multiple clinicopathologic factors (comprising age, pathological stage, and TNM stage) were effective predictors of survival in patients with BRCA. We assessed the Kaplan-Meier survival curve to verify these results. According to the survival curve, patients with an age > 60, distant metastasis, lymph node metastasis, tumor diameter > 5 cm, and pathological stage III–IV had a poor prognosis (Figure 9). This result further confirmed the reliability of our analysis.

**Figure 10:**
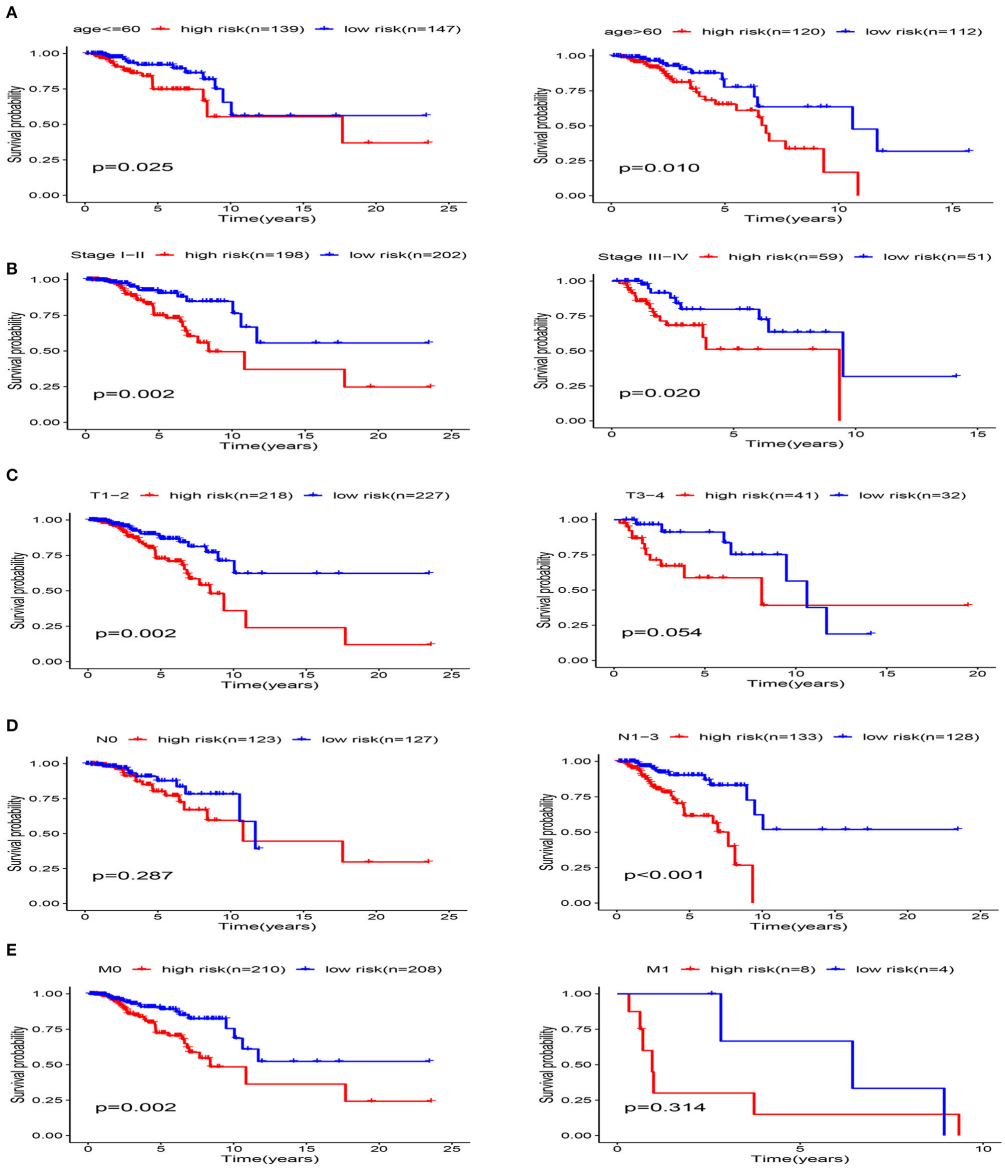
Stratification analysis of various clinicopathological factors using Kaplan-Meier curves for patients with BRCA in the training cohort. **(A)** Age, **(B)** pathological stage, **(C)** T Stage, **(D)** N Stage, **(E)** M Stage.

The clinicopathological parameters of BRCA patients in the training cohort are shown in Table 2. Next, we conducted a stratified analysis of age, pathological stage, and TNM. The patients were divided into age > 60 subgroup, age ≤ 60 subgroup, Stage I-II subgroup, Stage III-IV subgroup, T1-2 subgroup, T3-4 subgroup, N0 subgroup, N1-3 subgroup, M0 subgroup, and M1 subgroup. We found that in the stratified analysis, the survival time of patients in the high-risk group was significantly shorter than that of patients in the low-risk group, except for the M1 subgroup and N0 subgroup (Figure 10).

**Table 2:** Clinicopathological parameters of 519 breast cancer patients in the high and low risk groups in the training cohort.

**Characteristics**	**Riskscore**	
	**High-risk group**	**Low-risk group**
**Age**		
≤60 years	139	147
>60 years	120	112
**Stage**		
I	36	62
II	162	140
III	51	48
IV	8	3
Unknown	2	6
**Pathologic T stage**		
T1	59	86
T2	159	141
T3	29	28
T4	12	4
**Pathologic N stage**		
N0	123	127
N1	97	87
N2	22	24
N3	14	17
Unknown	3	4
**Pathologic M stage**		
M0	210	208
M1	8	4
Unknown	41	47
**Estrogen receptor**		
Negative	74	40
Positive	173	206
Unknown	12	13
**Progesterone receptor**		
Negative	107	62
Positive	141	184
Unknown	11	13
**HER2**		
Negative	118	139
Positive	58	23
Unknown	83	97

## Discussion

Abnormal glucose metabolism is considered to be a marker of tumors. The increase in aerobic glycolysis provides tumor cells with ATP and nutrients, while forming an acidic microenvironment, which is conducive to the mutation, metastasis, and invasion of tumor cells (Peppicelli et al., 2014). However, the prognostic significance of glycolysis-related genes in breast cancer has not been reported. In our study, we searched for biomarkers related to glycolysis for use in patients with BRCA. We used GSEA to identify mRNAs associated with glycolysis. BRCA samples were randomly divided into the training cohort and testing cohort, and then, the training cohort was used to perform univariate and multivariate Cox regression analysis to construct a BRCA prognostic risk signature. The specificity and sensitivity of this signature were verified in entire cohort and testing cohort. We found that a glycolysis signature composed of four glycolysis genes *(PGK1, SDHC, PFKL*, and *NUP43*) could predict the survival rate of patients with BRCA. Interestingly, in the hierarchical analysis, we found that the four-gene signature could also accurately predict survival, except in the MI subgroup and the N0 subgroup. The reason why positive results were not obtained in the M1 subgroup and N0 subgroup might be that the sample sizes were too small, and further analysis is needed.

Phosphoglycerate kinase 1 (PGK1) catalyzes the conversion of 1,3-diphosphoglycerate and ADP into 3-phosphoglyceride and ATP, respectively, and it is the first enzyme in the glycolysis pathway to produce ATP (Li et al., 2016). *PGK1* is not only a metabolic enzyme, but also a protein kinase, and it plays an important role in the occurrence and development of tumors (Fu and Yu, 2020). Previous researches have shown that the expression of *PGK1* is closely associated with BRCA. *PGK1* promotes the progression and metastasis of BRCA by adjusting the HIF-1α-mediated process of breast cancer epithelial-mesenchymal transition (Fu et al., 2018). Furthermore, the expression of *PGK1* is significantly associated with poor prognosis and resistance to paclitaxel chemotherapy in breast cancer (Sun et al., 2015). *PGK1* has also been reported in other cancers. Ahmad et al. reported that PGK1 was overexpressed in colon cancer tissues and is correlated with colon cancer metastasis (Ahmad et al., 2013). Hu et al. (2017) found that *PGK1* was overexpressed in liver cancer cells and that *PGK1* acetylation promoted its enzyme activity and cancer cell metabolism. Additionally, *PGK1* is also abnormally expressed in a great deal of cancers, such as gallbladder cancer (Lu et al., 2015), astrocytoma (Yan et al., 2012), gastric cancer (Zieker et al., 2010a,b), and pancreatic ductal adenocarcinoma (Hwang et al., 2006), and it is closely correlated to tumor occurrence, metastasis, and invasion. Moreover, *PKG1* can also be used as a target gene of miRNAs to modulate tumor development and progression. *Mir-548c-5p* may play an anti-cancer role by targeting *PGK1* to inhibit the generation of inflammatory cytokines and proliferation in colorectal cancer cells (Ge et al., 2019). *MiR-450b-3p* is underexpressed in liver cancer and at least partly through inhibiting *PGK1* to play a tumor suppressor effect (Chen et al., 2019). In addition to cell metabolism regulation, *PGK1* is also participate in a variety of biological activities, such as DNA repair, autophagy, and angiogenesis (He et al., 2019). Thus, *PGK1* is a very promising target for cancer treatment.

Succinate dehydrogenase (*SDH*) is a marker enzyme of mitochondria, which participates in the electron transport chain and Krebs cycle (Dalla Pozza et al., 2020); it is one of the hubs connecting oxidative phosphorylation and electron transfer. The loss of *SDH* function is the driving mechanism of many cancers (Killian et al., 2014). *SDH* germline mutations are associated with hereditary tumors such as renal cell carcinoma (RCC), pheochromocytoma (PC), paraganglioma (PGL), gastrointestinal stromal tumor (GIST), and pituitary adenoma (MacFarlane et al., 2020). Succinate dehydrogenase subunit C (*SDHC*) is one of the four subunits of *SDH*. Ishii et al. have shown that *SDHC* mutations increase oxidative stress and produce excessive ROS, thereby leading to nuclear genome mutations and cell transformation, which in turn leads to tumorigenesis (Ishii et al., 2007). The downregulation of *SDHC* in breast cancer promotes epithelial to mesenchymal transition and reconstructs the structure of mitochondrial organelles (Røsland et al., 2019). The epigenetic inactivation of the *SDHC* gene locus may be the cause of tumorigenesis in patients with Carney triad (CT) (Haller et al., 2014). Li et al. (2019) found that the activity of *SDH* decreased after *SDHC* knockout, thereby promoting the growth and metastasis of hepatocellular carcinoma cells through ROS/NF-κB signaling. Phosphofructokinase (*PFK*) catalyzes the conversion of fructose 6-phosphate and ATP into fructose 1,6-diphosphate and ADP, and it is a key enzyme in glycolysis (Lee et al., 2017). The *PFKL* gene is a *PFK* subtype. Yang et al. (2016) have reported that the Mir-128-PFKL-Akt axis promotes the proliferation and independent growth of lung cancer cells by regulating glycolysis. *PFKL* rs2073436C>G can be used to predict the clinical efficacy of first-line paclitaxel-cisplatin and cisplatin therapy for NSCLC (Choi et al., 2020). The upregulation of *NUP43* is associated with DNA amplification, which can independently predict the overall survival rate of lumen A and HER2+ breast cancer (Tian et al., 2018). *MiRNA-409-5p* inhibits proliferative potential and cell cycle progression in child chronic myeloid leukemia (CML) by upregulation of *NUP43* expression (Liu et al., 2019).

In the KEGG enrichment analysis, we found that three genes (*PGK1, SDHC*, and *PFKL*) in the signal were enriched in the carbon metabolism pathway. Carbon metabolism is one of the most fundamental aspects of life. Glycolysis is the core pathway of cell carbon metabolism, and it not only produces ATP to provide energy but also provides biomass to promote cell proliferation (Yan et al., 2019). Central carbon metabolism includes the carbon utilization pathways of glycolysis, the citrate cycle, and the pentose phosphate pathway. Many diseases have changes in central carbon metabolism, including cancer, diabetes, and heart disease (Walsby-Tickle et al., 2020). Schraw et al. (2019) showed that changes in central carbon metabolism and amino acid metabolism were related to minimal residual disease (MRD) positivity and that metabonomics has potential utility in the risk prediction and targeted treatment of pediatric acute lymphoblastic leukemia (ALL). In HNSCC, cisplatin-induced oxidative stress triggers rapid changes in carbon flux in central carbon metabolism, which can provide potentially useful information for predicting treatment response (Yu et al., 2018).

We constructed a four-gene risk signature based on glycolysis using the RNA expression and clinical information on patients with BRCA available in TCGA database that can be used to accurately predict the prognosis of patients with BRCA. Additionally, ROC curve, univariate, and multivariate Cox regression analyses combined with clinical information (such as age and TNM) showed that the four-gene signature had high specificity and sensitivity for prognosis, which further demonstrated the evaluation value of the signature. However, the present research had some limitations. This predictive signature requires further clinical and experimental verification to improve its accuracy. We also tried to verify the predictive ability of the four-gene signature in other databases, but unfortunately, because of the limitations of patient clinical information, we could not find a suitable data set. In summary, our research outcomes indicate that the glycolysis-related four-gene signature will help to open new avenues for the development of breast cancer treatment strategies, and it may aid in efforts to achieve individualized treatment.

## Conclusion

We developed a glycolysis-related four-gene signature to predict the prognosis of patients with BRCA. Although the mechanism of action of these mRNAs in breast cancer needs to be confirmed, the signature we established provides theoretical support for the use of bioinformatics in evaluation of patient prognosis.

## Data Availability Statement

Publicly available datasets were analyzed in this study. This data can be found here: The data that support the findings of this study are available in The Cancer Genome Atlas database at https://cancergenome.nih.gov/.

## Author Contributions

XZ and JW designed research topic. JZ, XM, and CL obtained data on Breast cancer. CG, JL, and HL build the related network and analyze the data. XZ and JW wrote the manuscript. CL, CS, and CG revised the manuscript. All authors read and approved the final manuscript.

## Conflict of Interest

The authors declare that the research was conducted in the absence of any commercial or financial relationships that could be construed as a potential conflict of interest.
